# Construction of environmental vibration prediction model for subway transportation based on machine learning algorithm and database technology

**DOI:** 10.1038/s41598-024-56940-3

**Published:** 2024-03-15

**Authors:** Xilong Zhou

**Affiliations:** Mechanical and Industrial Engineering Department, College of Engineering, Northeastern University, 360 Huntington Ave, Boston, MA USA

**Keywords:** Ambient vibration in subway transportation, Accuracy, Machine learning, Database, Prediction, Mechanical engineering, Applied mathematics

## Abstract

Vibrations generated in the metro transport environment are mainly caused by, vibrations generated by the interaction between the metro and the track during operation. and the change of vibration factors will affect the normal operation of the subway. However, it is difficult to have a model that can achieve the characteristics of high accuracy, fast computing speed and wide range of use in the traditional metro rail transportation environment prediction. Therefore, this research uses database theory and machine learning algorithms to predict the vibration of subway transportation environment. The experimental results show that the average difference between the whole prediction value and the real value is 1.4 dB, of which the maximum difference error value is 0.29%, the maximum error difference is 8.2%, and the approximate value is 6.2 dB, and the four averages predicted in 40 m are relatively small as 1.6 dB, and the average error value of prediction ability between 40 and 100 m is 1.72 dB, and the experimental prediction value and real value are in good agreement. The agreement between the experimental prediction and the real value is very good. Therefore, the model is able to predict the vibration model of the subway transportation environment with a high degree of agreement and accuracy.

## Introduction

At present, with the development of urban transportation, more and more public transportation has become the first choice for people's daily travel, and subway transportation has also become the first choice for current public transportation^1^. In the process of subway transportation construction, many factors will affect the impact of subway construction, such as the building environment, soil layer, track vibration, etc., in which the subway track vibration is the hot topic of many current studies, how to achieve accurate prediction of the subway transportation environment has become an important research direction for the study of subway transportation environment^2^. Database technology is the basic theory used in data management and computation, which can be widely used in the management and storage of data^3^. Machine learning algorithms are commonly used computer algorithms that can analyze and predict some complex data^4^. In the traditional subway traffic environment vibration prediction, many prediction models cannot simultaneously meet the requirements of high accuracy, fast calculation speed and wide range of use, so many experts and scholars have done a lot of research on it. Among them, Ling, Yuhong et al. believe that the vibration of the track will affect the construction and construction when building a subway, so in order to predict the amplitude of vibration during the construction, a three-dimensional model composed of soil, load and building was constructed by using a new model. The new model was able to parameterize the construction process. Through experimental verification, it was proved that the new model was able to assess and predict the subway vibration, and also the applicability of the new model was high^5^. Dai Chunquan et al. believed that the traditional subway construction process without predicting the vibration situation may cause the subway train collision, so after the study of the three-dimensional model of the train track, an ABAQUS-based Subway track model, the new model can detect the centerline and center distance of the railroad track to achieve the prediction of ground parameters. The experimental results show that the new model can reach the ideal situation for the prediction of both track loads and track parameters^6^. Wang Lidong et al. Therefore, in the prediction of track vibration, a new stochastic prediction method was built using a combination of a two-dimensional system and a multidimensional model, and the new method is able to analyze and predict the data of the track's support and defects. The experimental results show that the new method can improve the computational efficiency, while the feasibility of the method has also been proved^7^. Liu Shaowu believes that with the development of the train subway, the vibration problem of the subway environment is becoming more and more prominent, therefore, in the study of subway tunnels, the internal data of the tunnel's building were collected, and a new indicator comfort evaluation program was proposed. The scheme is able to predict the noise and vibration situation of the subway vibration environment, and reduce the impact of vibration and noise. The experimental results show that the noise of the subway operation after using this scheme is significantly reduced, and the building vibration are reduced^8^.

Huang Hong-Yuan et al. believe that in the study of subway tunnel vibration, the study of track concrete will affect the vibration situation, so in the study of subway traffic vibration needs to take into account the state of concrete, which establishes a vibration model for the study of the characteristics of the concrete material foundation, the new model can be studied and analyzed on the concrete structure to achieve the prediction of vibration conditions. The experimental results show that the new model is reasonable for the prediction of the condition of the subway environment construction^9^. Sheng Tao et al. believe that improving the subway vibration human comfort can reduce the subway vibration. Therefore, in the study of subway vibration, increasing the vibration isolation effect of the mid-rise building can enhance the impact of vibration on the subway. The experimental results show that the use of different sandbags to reduce vibration is easy to realize for many buildings^10^. Lidong Wang et al. proposed an efficient time–frequency method in order to predict tunnel and ground vibrations induced by subway trains. The new method has two steps firstly to determine the track-tunnel interaction forces by simulating the vehicle-track subsystem in the time domain, and secondly to apply the derived forces to a 2.5D FEM-PML model of the tunnel-soil system. The results show that the linearized Hertz wheel-rail contact model has a relatively small error of less than 2% in calculating the track-tunnel interaction force by comparing it with the nonlinear Hertz contact model^11^. Ahmed A Khalild et al. In order to analyze the effect of rubber mat system on vibration levels, stress values and deformation of the metro tunnel section, a three-dimensional model was created in ANSYS program for finite element analysis. The proposed track system in the tunnel of Metro Line 4 in Greater Cairo was used as a case study to analyze the effect of the stiffness of the rubber mat system on the vibration level. The results show that the vibration level is logarithmically related to the stiffness of the rubber mat system, and the stiffness also has a significant effect on the deformation and stress^12^. Jia-Hua Yang et al. proposed a new two-stage Markov chain Monte Carlo method in order to solve the problem of uncertainty in building vibration data, the new method searches through the parameter space and the data probability data of building vibration points. The results of the study show that the use of the new method can improve the effect on the building vibration^13^. Santos et al. In order to better planning and dynamic response of the route of the railway, a new experimental method of a new underground vibration behaviour data model is proposed, the new method by simulating the dynamic data behaviour of the underground, which in turn projects the new underground vibration information data. The results show that the use of the new method can simplify the dynamic data model of underground vibration and improve the ability to assess the underground vibration model^14^. Wang et al. proposed a new time–frequency stochastic prediction method for the current problem of predicting the tunnel vibration and ground vibration caused by the underground train, and the new method simulates and analyses a two-dimensional multi-body system by using the pseudo-excitation method. A new track irregular power density is also used to derive the interaction force of the metro track. The results of the study show that the use of the new method can effectively improve the data prediction simulation of underground vibration conditions^15^.

In summary, many experts and scholars in the vibration of the subway environment research, got a lot of research results, at the same time in the study found that some other factors change will affect the subway vibration situation, many of the current models still have a lot of problems, such as the lack of accuracy of the current prediction model, the use of the range of small and other issues. The current study found that the factors affecting the underground vibration is complex, underground vibration will be affected by different degrees of environmental impact, while the underground data prediction effect of the current use of methods there are many shortcomings, for the underground traffic environment vibration data model and data analysis methods are still less, so the current existence of the study there are still many shortcomings. Therefore, for the vibration of the underground environment, the need to improve the accuracy of the model prediction and use of the scope, as well as the ability to analyse the vibration data. so this study proposes the prediction of subway track model based on machine learning database, used to achieve the model prediction of the accuracy and speed of calculation to improve. The database can also be used to analyze and store more data about the subway transportation environment. Therefore, by embedding the machine learning algorithm into the database system, a new machine learning algorithm of underground traffic environmental vibration database prediction model is built, so as to achieve the efficient prediction of underground traffic environmental vibration. This study is divided into five parts, the first part outlines the research at home and abroad, and briefly introduces the shortcomings and advantages of some studies; the second part establishes a vibration prediction model using machine learning algorithms and databases; the third part verifies the accuracy and prediction precision of the model using the experimental results; the fourth part carries out an in-depth analytical discussion of some of the reasons for the experimental results; and the fifth part summarizes the full text and points out the direction of the subsequent research.

## Construction of track vibration model and algorithm design based on machine learning algorithm and database technology

This chapter focuses on a brief overview of the prediction methods that need to be used and the construction of the methods, and then through the creation of a database, a new model for predicting track vibration in metro transportation using machine learning algorithms was constructed. The new model combines machine learning algorithms and a database model, enabling it to be more conducive to the analysis and prediction of metro rail data.

### Database theory and machine learning algorithm theory research

**Figure 1:**
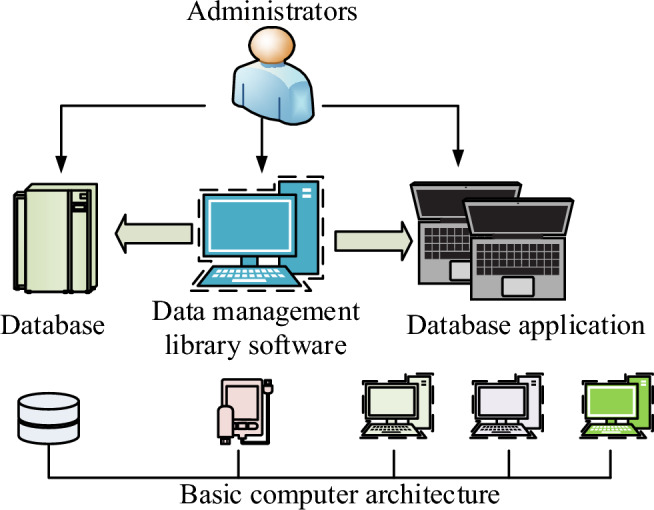
Basic database framework.

Database is a general term for the entire database system, which includes the entire system services, program services, librarians and computer applications. Which the construction of the database is generally on some logical language and information parameters for the design, usually use the design language for the SQL, mainly using the computer for the construction of the database as shown in Fig. 1 for the database of the basic system construction model^16^.

**Figure 2:**
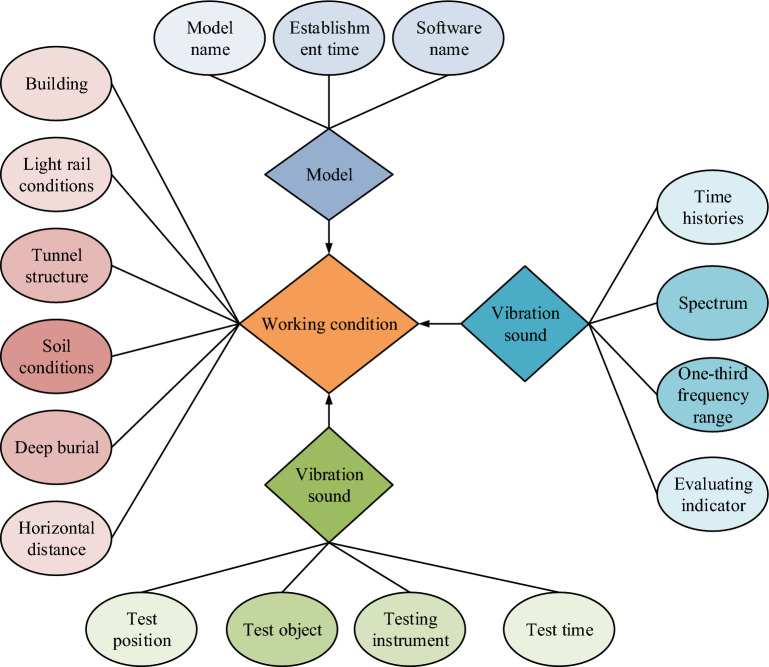
E–R model basic structure.

The management system in the management system of the database in Fig. 1 includes the relevant collection in the database, the software that manages the database, the relevant programs in the database, etc. are unified by the data administrator, and the user information and parameter storage are saved by the computer structure. Database through the maintenance of data management and control, used to achieve the management and storage of data. In the construction of the database, it is necessary to analyze the demand, determine the target data needed, and determine the information and entity information to be stored. The data prediction of the subway traffic environment is needed to store the subway environmental traffic and environmental vibration data, the database mainly serves the vibration measurement data and numerical calculation data^17^. In the construction of the data structure, including several kinds of storage information of the database, construction information, data detection information, vibration information, model building information and so on. Construction information is a collection of structure, soil, level, distance and building information for subway track information; vibration information and modeling information are a collection of actual information data on site. In the design of the database usually use the E–R model for model building, the E–R model is a conceptual method model that can describe the data of the real world. It is shown in Fig. 2.

As shown in Fig. 2, the total construction conditions include a summary of the tunnel structure and soil conditions, which also includes a summary of the construction distance construction depth, the basic simulation model and vibration conditions are summarized in the construction conditions, in which the vibration conditions need to determine the vibration frequency, spectrum, and evaluation indexes, and experimental testing needs to be determined for the target, location, and instrumentation time and so on. The design of the model requires the use of software to build the model time name.

**Figure 3:**
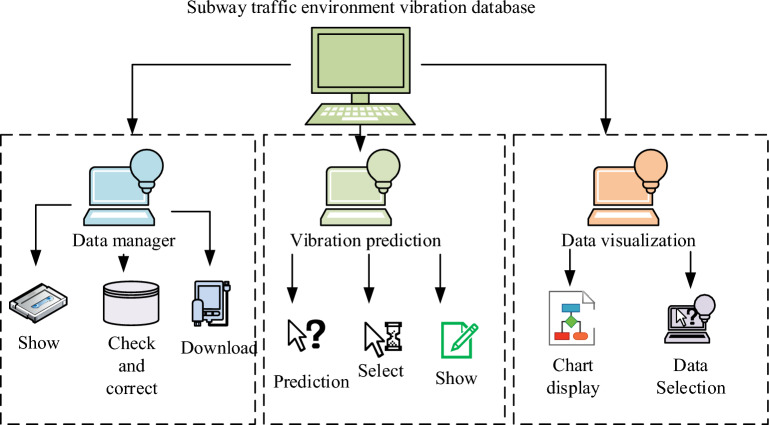
Database function diagram.

In the subway traffic environment of the model for the experiment, the need for managers to manage the database, data mobilization, the state of the vibration of the state of the prediction and other state of the analysis, for the management of data is mainly on the subway traffic environment vibration of the actual measurements and numerical data to manage the query and call. Vibration prediction is mainly on the subway better environmental vibration field using machine learning algorithms for data learning, so as to simulate the working conditions of the training and prediction, data visualization is mainly the use of charts and graphs of information on the data to analyze and display^18^. As shown in Fig. 3 the various functions of the database are introduced.

As shown in Fig. 3, the system components of the database are mainly data management, vibration model predictive analysis, and visualization and analysis of data. Through the analysis and use of the three modules can realize a variety of functions of the database, so as to then use learning algorithms to calculate and analyze the data. Machine learning is a learning method that allows computers to learn through data by issuing instructions to the computer, thereby commanding the computer to perform operations such as programming, and traditionally there are a variety of artificial learning algorithms, including decision trees, clustering, Gaussian, and vector machines. Deep learning then belongs to a sub-domain of machine learning learning algorithms, belonging to a more detailed division of machine learning in the collection. For the analysis of vibration in the underground traffic environment, it is necessary to take the parameter information of the train, such as speed, depth of vibration source, horizontal distance, shear wave velocity, damping ratio, and other factors affecting the vibration response of the subway traffic environment as the sample features, and the response parameters of vibration as the dependent variables. Therefore the parameter analysis is carried out using deep learning algorithms in machine learning algorithms, but since neural networks consist of a large number of neurons capable of transmitting and processing data information, the neurons are also capable of being trained and strengthened into a fixed neural ideology that allows for a stronger response to specific information. This allows for better data analysis and prediction of vibration situations in the metro traffic environment, which suggests that using neural network models is a better method of model prediction and analysis. Among them, for the traditional machine learning algorithm model building is mainly divided into input layer, output layer and hidden layer. Among them, the formula for the input layer is shown in Eq. (1)^19^.

1 $$x_{i} = a_{i}$$

In Eq. (1), $x_{i}$ denotes the value output to the next layer and $a_{i}$ denotes the input data. Where the hidden layer is calculated as shown in Eq. (2).

2 $$y_{j} = \sum\limits_{i = 1}^{M} {w_{ij} x_{i} }$$

In Eq. (2), $y_{j}$ denotes the output value of the input to the next layer and $w_{ij}$ denotes the weights between the input layer and the hidden layer. The formula for the input layer is shown in Eq. (3).

3 $$o_{k} = \sum\limits_{j = 1}^{o} {w_{kj} s_{j} }$$

**Figure 4:**
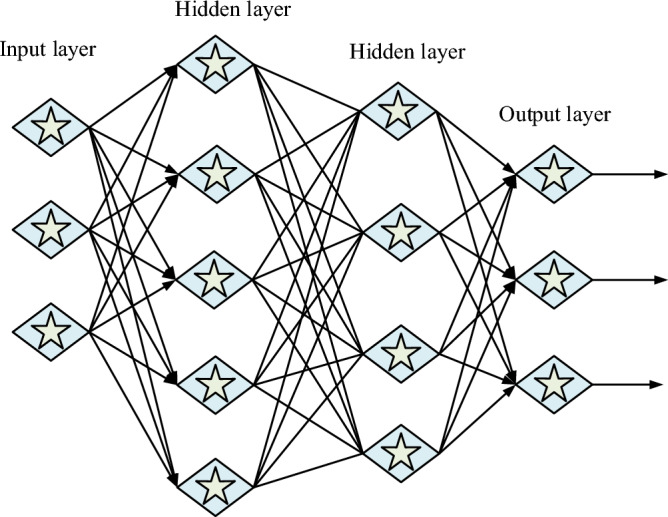
Neural network structure of machine learning algorithm.

In Eq. (3) $w_{kj}$ denotes the weights between the hidden layer and the output layer, and $s_{j}$ denotes the value of the output change through to the hidden layer, where $s_{j} = f(y_{j} )$. Where the number of samples set is $m$, the number of neurons in the neural network is $M$ and the number of hidden layers is $Q$^20^. The basic model of the machine learning algorithm obtained after calculating each value is shown in Fig. 4.

In Fig. 4, the basic structure of the machine learning algorithm consists of neuron visualization, where the input layer represents the input of data and passes it to the next layer, and the hidden layer represents where the data is transferred to the next output layer through computation and analysis. The output layer is the input and processed data through here to complete the final model data output. After the completion of the construction of the machine learning algorithm, it is necessary to calculate some of the necessary data such as some of the basic subway units within the railroad track control equations. As shown in Eq. (4).

4 $$E_{r}^{*} I_{r} \frac{{\partial^{2} u_{r} }}{{\partial x^{4} }} + m\frac{{\partial^{2} u_{r} }}{{\partial t^{2} }} = Fe^{iw0t} \delta (x - \overline{{x_{0} }} - vt)$$

In Eq. (4), $E_{r}^{*}$ represents the material and damping change elastic model of the railroad track, $v$ represents the moving speed when loading, $w$ represents the number of angular rotations of the track, $u_{r}$ represents the moving distance of the track when it is in vertical direction, $m$ represents the mass of the track per unit length, $\overline{{x_{0} }}$ represents the change of the train's position in the initial moment of loading, and $x$ represents the moving distance. The damping force formula for the rail is shown in Eq. (5).

5 $$E^{*} = E(1 + 2i\beta )$$

In Eq. (5), $\beta$ denotes the ratio of damping in the medium, $E$ denotes the modulus of elasticity, and $E^{*}$ denotes the modulus of elasticity after considering the damping of the medium. As shown in Eq. (6)^21^.

6 $$G^{*} = G(1 + 2i\beta )$$

**Figure 5:**
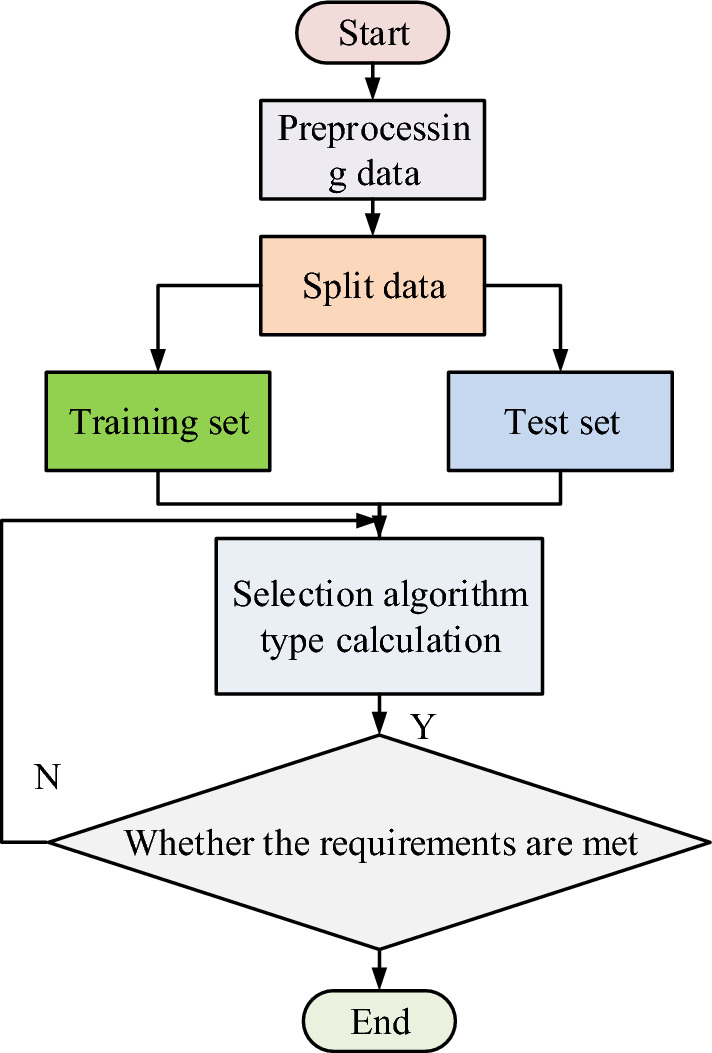
Basic operation process of machine learning algorithm.

In Eq. (6), $G$ denotes the shear modulus and $G^{*}$ denotes the shear modulus after considering the medium damping. The two formulas are able to calculate the track operation data in the subway environment, thus obtaining the prediction data and experimental data that need to be calculated by the machine learning algorithm. As shown in Fig. 5 is the flow chart of the basic operation structure of the machine learning algorithm^22^.

As shown in Fig. 5, first of all, in the machine learning algorithm on the subway environment track data to start the phase, the need to first initialize the data processing, by preparing the data set will be split into the data set, split into the training set of a test set of two parts, respectively, and then the algorithm of the data set, in the calculation of the data set of different data using the appropriate machine learning algorithms to analyze the data set, and train the appropriate algorithms after the analysis. At the same time, the size and features of the training algorithm are obtained through the above split analysis. Finally, the model is then used to determine whether the data set needs to be analyzed and calculated to output the algorithm model^23^.

### Construction of environmental vibration prediction for metro transport based on machine learning and SQL database

The use of machine learning algorithms and database technology in the prediction of the subway transportation environment can improve the accuracy of the prediction data and reduce the cost of prediction by predicting the construction program, drawings, operation period and program enforceability data. Algorithmic predictive modelling applications for databases are generally programmed in Python. By saving the data information as a .py file in the Python programme, the entire data information is a single Python module, which can be imported into a different application using the import module in the programme. Therefore saving the algorithmic model and then calling the new data algorithmic program through the program ensures that the current data information can be used by the algorithmic model, which in turn enables the conduction of data between the model predictions and the database.

For the construction process of the algorithmic model and the underground vibration SQL database, firstly, in the query of the data parameters of the underground traffic environment, send data prediction requirements; SQL database through the search engine will get the data transmission into the server, after receiving the data through the Python programme view tool will be converted to the data module, and then the data will be transmitted into the algorithmic prediction model, and secondly Receive the current prediction result parameter data, and finally display the front-end interface in the form of data conversion in the Python programme to get the final prediction result data. At this time to complete the construction process of the database algorithm prediction model.

Vibration modeling refers to the use of formulas and measurements of vibration targets along the construction line to simulate the speed vibration frequency and vibration size of the vibration targets. However, the prediction results using the model need to make reference to the subway construction environment value and significance^24^. The traditional subway vibration prediction model uses the construction of empirical prediction methods, experimental prediction methods and other methods. The empirical prediction method is the more widely used prediction method, often using the fitting of the formula to achieve the vibration target prediction of the subway environment. As shown in Eq. (7).

7 $$L_{a} (room) = L_{t} (tunnelwall) - C_{g} - C_{gb} - C_{b}$$

In the formula (7), $L_{a} (room)$ indicates the acceleration level of the inner wall of the tunnel in the construction of the subway building, and $L_{t} (tunnelwall)$ indicates the vibration acceleration level of the ground in the construction of the subway. $C_{g}$ $C_{gb}$ and $C_{b}$ indicate the attenuation of vibration in the vibration fault, vibration into the tunnel, vibration in the tunnel. Equation (8) for the prediction of vibration noise and vibration sound pressure formula.

8 $$L_{B} = L_{r} + R_{tr} + R_{tu} + R_{g} + R_{b}$$

In Eq. (8), $L_{B}$ denotes the predicted sound pressure level of the subway environment, $L_{r}$ denotes the velocity level of the subway track, $R_{tr}$ denotes the amount of vibration energy lost in the subway track, $R_{tu}$ denotes the reduction of vibration energy transmitted in the subway channel, $R_{g}$ denotes the energy lost in the transmission of vibration energy at the ground level, and $R_{b}$ denotes the loss of vibration energy in the inner wall of the subway tunnel. The vibration prediction formula when the subway passes through the soft soil layer is shown in Eq. (9)^25^.

9 $$V = F_{v} F_{R} F_{B} = [V_{T} F_{S} F_{D} ]F_{R} F_{B}$$

In Eq. (9), $F_{v}$ represents the change of the function of vibration in the subway channel, $F_{R}$ represents the change of the mass of the subway track, $F_{B}$ represents the change of the subway building after amplification, $F_{T}$ represents the type of the train passing through the subway, $F_{S}$ represents the speed of the train, and $F_{D}$ represents the distance of the train. The vibration prediction data can be obtained by the method of hammer tapping. As shown in Eq. (10)^26^.

10 $$L_{v} = L_{F} + TM_{line} + C_{building}$$

In Eq. (10), $L_{v}$ denotes the vibration velocity level of the predicted value, $L_{F}$ denotes the force density level of the vibration generating source, $TM_{line}$ denotes the linear transmission efficiency of the train vibration, and $C_{building}$ denotes the corrected energy of the vibration transmitted from the ground to the inner wall. The formula for the change of vibration environmental impact during train operation is shown in Eq. (11).

11 $$VL{}_{Z\max } = VL_{Z0\max } + C_{VB}$$

In Eq. (11), $VL{}_{Z\max }$ denotes the predicted maximum vibration level, $VL_{Z0\max }$ denotes the predicted vibration source intensity, and $C_{VB}$ denotes the correction index of the vibration target. The improved formula of Eq. (11) is shown in Eq. (12) .

12 $$C_{VB} = C_{V} + C_{W} + C_{B} + C_{T} + C_{D} + C_{B} + C_{TD}$$

In Eq. (12), $C_{V}$ represents the correction value of speed of running train, $C_{W}$ represents the correction value of bearing weight and spring mass of train, $C_{R}$ represents the correction condition value of train track, $C_{T}$ represents the correction value of different track styles, $C_{D}$ represents the correction value of attenuation of distance, $C_{B}$ represents the correction value of building in the project, and $C_{TD}$ represents the correction value of density of the train traveling. The change formula of vibration parameters in different areas is different, such as Eq. (13) for the vibration value in another area.

13 $$VL{}_{Z\max } = VL_{Z\max ,0} + C$$

In Eq. (13), $VL_{Z\max ,0}$ denotes the vibration level of the maximum measured vibration source of the train passing through the engineered tunnel, and $C$ denotes the corrected value of vibration.

14 $$C = C_{v} + C_{g} + C_{l} + C_{b} + C_{L} + C_{B}$$

Equation (14) is the expression of $C$ value, in Eq. (14), $C_{v}$ represents the correction value of the train's traveling speed, $C_{g}$ represents the mass of the train's bearings, $C_{l}$ represents the correction value of the train's traveling curve, $C_{b}$ represents the correction value of the train's track, $C_{L}$ represents the correction value of the train's traveling distance, and $C_{B}$ represents the correction value of the track's construction. The empirical method is able to calculate the fitted formula, so using the empirical method for prediction can make the prediction more effective and less costly, so using the empirical method for prediction during the construction phase of the subway will make the construction project less costly, but the lack of accuracy of the empirical method is a big problem of this method. At this time the use of machine learning neuron method can greatly reduce the construction cost and enhance the accuracy of prediction.

**Figure 6:**
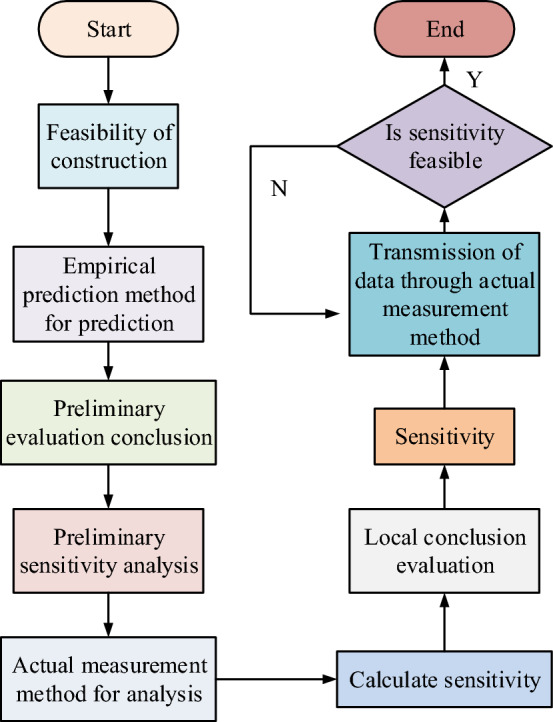
Prediction and evaluation of subway construction environment.

Test prediction is the vibration assessment of the actual predicted vibration targets in the field, meaning that the actual measurement of the field data is then predicted. However, in the actual field test, because the subway has not yet been completed, the test data results are mostly simulation results data. Hybrid prediction method is through the prediction results of the accuracy and multi-parameter system coupled to the different methods of simulation experiments so that the experimental prediction method for the hybrid prediction method, usually hybrid prediction method can solve the complex parameter uncertainty and multi-data mixed system error problems. As shown in Fig. 6, the prediction and evaluation process of subway construction environment.

Figure 6 shows that in the feasibility of the program stage needs to be the subway line and construction phase of the feasibility analysis, through the empirical prediction method of the subway environment model for the prediction of the parameters, to achieve a full range of environmental prediction of the preliminary assessment. After the predictive analysis of the feasibility of the program, the actual data of the subway environment using the actual method of testing and analyzing the sensitivity of the calculator environment, to achieve the local data prediction of the subway environmental parameters. In the construction design stage, the sensitivity is judged by using the actual testing and in-tunnel measurement method to determine whether the sensitivity meets the construction requirements. Finally, the total construction process evaluation conclusion is calculated. In the prediction model of machine learning and database technology, the selected characteristic parameters include column velocity, vibration depth, distance, damping ratio, density, Poisson's ratio, shear wave speed and other influencing factors. Therefore, multiple neuron parameters need to be designed while building data for the model. The number of neurons designed determines the number of parameters selected for the data, the more parameters for the experimental data will be more comprehensive, and the whole prediction results will be more accurate.

## Analysis of subway transportation environmental vibration prediction results based on machine learning algorithms and database technology

**Figure 7:**
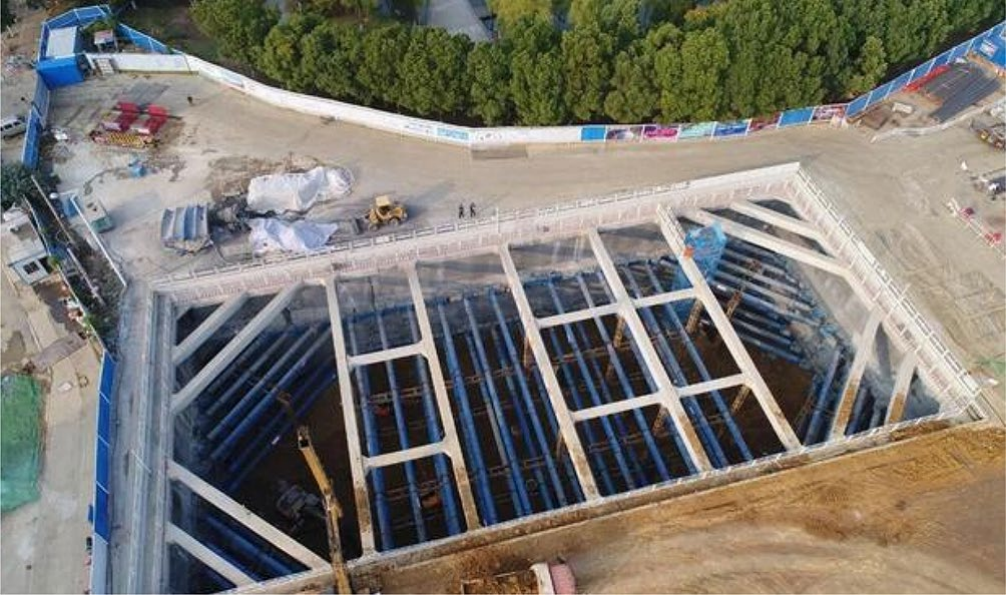
Construction site plan.

Experimental test site selection of a construction site, the current construction site is not under construction, the ground is relatively flat, away from public transportation, no buildings in the surrounding 150m, as shown in Fig. 7 for the building construction site map.

Meanwhile the study in the track composition of the tunnel spacing size of 14.5m, the size of the outer diameter of 4m, the height of the vault is 10m, the track using ballasted track. The database parameters used in the study were tested for actual metro parameters obtained as shown in Table 1.

**Table 1:** Information on measured data in the database.

Condition number	Actual measurement situation	V	H	Cs	Ds	p	u	l
1	Group 1	40	22	290	0.03	1950	0.3	45
2		40	22	290	0.03	1950	0.3	52
3		40	22	290	0.03	1950	0.3	61
4	Group 2	45	20	386	0.03	2050	0.26	24
5		45	20	386	0.03	2050	0.26	26
6		45	20	386	0.03	2050	0.26	27
7		45	20	386	0.03	2050	0.26	34

As can be seen in Table 1, the measured data from the database in the study are actual measurements of the current train speed of the metro, the depth of the vibration source, the horizontal distance of the metro, the vibration shear fluctuation velocity of the metro, the damping ratio of the metro, the Poisson's ratio, and the density of these parameter data. In the study, this experiment uses the orthogonal test method to test the values and working conditions, orthogonal test is a multi-element and multi-level test design method. It is also able to reduce the calculated values and workload. The range of analysis, elastic modulus of the vibration source, damping ratio, density, Poisson's ratio, and shear velocity are analyzed for different six working conditions. The variation of parameters for different soil layers is shown in Table 2.

**Table 2:** Element changes in different soil layers.

Name	Solum1	Solum2	Solum3	Solum4
Range (m)	2.9	9.5	5.1	\windshield
Dynamic modulus of elasticity (E/Pa)	0.8	2.9	4.8	3.5
Poisson’s ratio	0.3	0.3	0.3	0.3
Damping ratio	0.03	0.03	0.03	0.03
Density (ρ/(kg/m^3^))	1800	1800	2000	1950
Shear wave velocity (Cs/(m/s))	150	200	290	300

**Figure 8:**
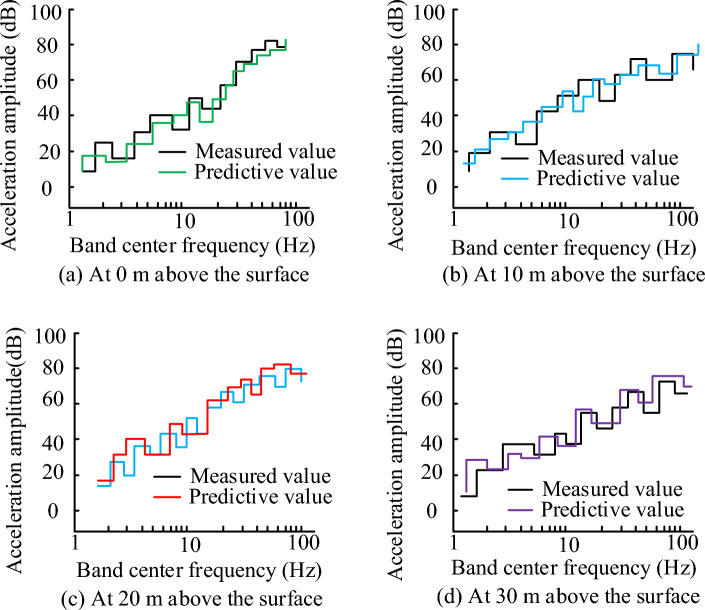
Comparison of measured and predicted data at different surface depths.

As shown in Table 2, the damping ratio and Poisson's ratio of different soil layers are the same, but the measurement range in soil layer 4 is in the state of unmeasured, and the measurement range of the rest of the soil layer is below 10, but from the table, it can be seen that the measurement range and the parameter changes are not directly related to each other, in which the elastic modulus of the largest value is in the soil layer 4, and the magnitude of the density of the soil layer influences the wave velocity of the shear when the density is large when the wave velocity of the shear is larger. Therefore, the variation of the whole soil layer is more related to the density, but the influence of other elements on the vibration of the soil layer exists. The feasibility of the prediction model is verified by experimentally testing the depth of the subway environment. As shown in Fig. 8.

**Figure 9:**
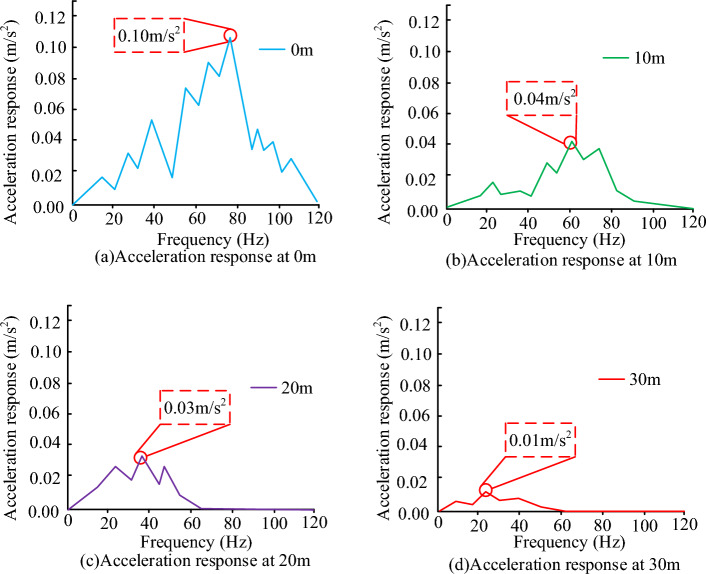
Acceleration response at different depths.

In Fig. 8, the measured and simulated values are compared for four different surface depths, in which the entire simulated and measured acceleration values change more when the frequency range increases within 10, and the acceleration amplitude instead tends to increase less when the frequency range exceeds 10. In the four different depths of the predicted amplitude, the predicted acceleration amplitude size and the actual amplitude size in the same centre frequency amplitude comparison, the amplitude of the largest difference is 15 amplitude, the smallest difference is 0 amplitude, the average amplitude difference in different depths are 3 amplitude of 0 m, 2 amplitude of 10 m, 9 amplitude of 20 m, and 8 amplitude of 30 m, which shows that the measured acceleration change and the prediction of acceleration changes in a high degree of agreement, and the measured acceleration change and predicted acceleration changes. and the acceleration amplitude is slightly smaller than the measured acceleration amplitude at the depth of 0 m. The possible reason for this is that there may be some unknown factors interfering at 0 m on the surface, but the overall prediction model is able to predict the vibration changes well. The train speed is set to $60\,\text{km}/\text{h}$, the construction depth is set to $30\,\text{m}$, the wave velocity to the soil layer is set to $230\,\text{m}/\text{s}$, the Poisson's ratio is set to $0.3$, the damping ratio is set to $0.03$, and the density of the soil layer is set to $1750\,\text{kg}/\text{m}^{3}$. The test results of the vibration response at different depths of the soil layer are shown in Fig. 9.

**Figure 10:**
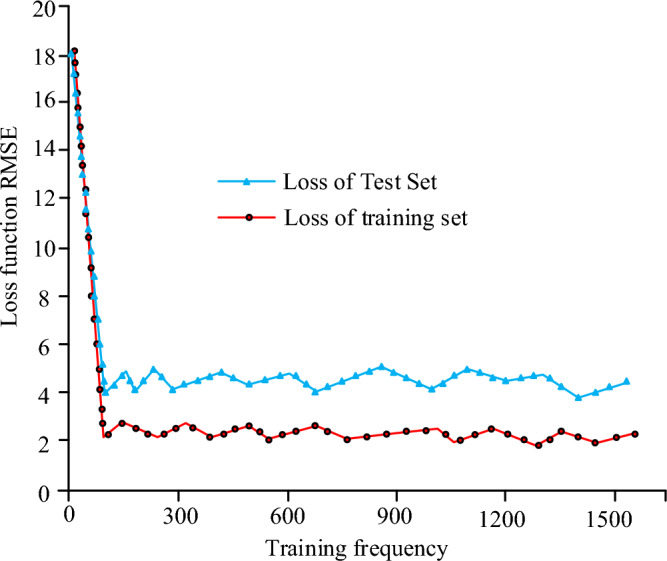
Loss function RMSE at different training fregquency.

As shown in Fig. 9, at a depth of 0 m to the surface, the acceleration response is higher overall where the highest value is at 0.1, with the increase in frequency, the value of the acceleration response slowly increases to reach the maximum value when it begins to show a downward trend. When the depth gradually increases the acceleration response value gradually decreases, where the acceleration response is the smallest at a depth of 30 m, the maximum acceleration value of four different depths are 0.1, 0.04, 0.03, 0.02. The possible reason is that the soil layer is too thick to accumulate resulting in the test of the value of the smaller. When experimenting on the feasibility of the algorithm, the number of neurons was set to 60, the number of training times was 1500, and the training set used was 200 sets, which resulted in the plots shown in Fig. 10.

**Figure 11:**
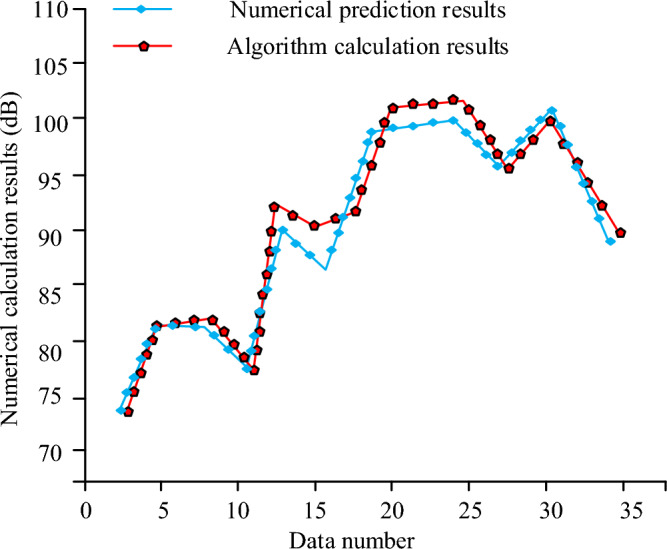
Algorithm prediction results and numerical calculation results.

As shown in Fig. 10, when the number of training times increases, the test loss value of the algorithm gradually decreases in the decline to the value of 4, the overall algorithm's loss function no longer decreases but with the change in the number of training times, there is a fluctuating state, but the overall value is always stabilized at about 4. The training loss value curve of the algorithm changes the same as the test value, but in the loss function value of the training set of the loss value is lower only about 2, the training set and the test set of the loss function deviation of 2. Through the vibration of the algorithm of the vibration data measurement to obtain the algorithm as shown in Fig. 11 measured comparison chart.

**Figure 12:**
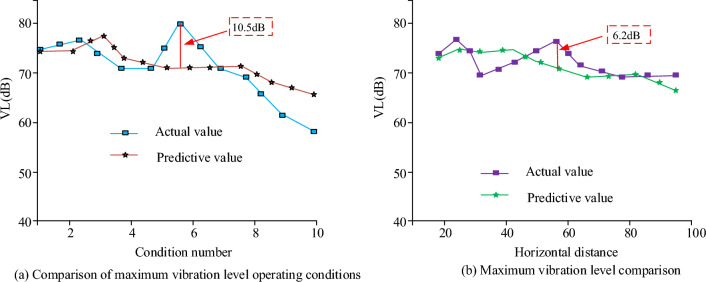
Comparison between predicted maximum vibration level and actual results.

Figure 11 shows that the algorithm prediction results and numerical calculation results curve changes are similar, basically using the algorithm prediction results and using the numerical will algorithm results are the same, which indicates that the use of the algorithm to predict the model to achieve the same effect and the actual measurement of the calculation. The curve reaches its maximum in the numerical calculation when the number of samples is 25, the value is 100 and the agreement of the algorithm is 92.57%. This shows that using the algorithm to predict the sample model can achieve the expected results. The largest vibration level in the working condition is predicted by the algorithm to obtain the prediction graph shown in Fig. 12.

In Fig. 12, in the comparison of the algorithm's prediction of several and the real value calculation results, the error is the largest in the working condition number 6, when the difference value is 10.5 dB, which may be caused by the existence of some operational problems during the measurement process, and the data existing in the database is not very good for the measurement. When analyzing the horizontal distance, the average difference between the whole prediction value and the real value is 1.4 dB, of which the maximum difference error value is 0.29%, the maximum error difference is 8.2%, and the approximate value is 6.2 dB. The overall coincidence is good, and the prediction effect is good. In the prediction of numerical prediction, the overall prediction of the four average values predicted in 40 m are relatively small 1.6 dB, between 40 and 100 m the prediction ability of the average error value of 1.72 dB, so in the prediction of vibration point prediction, the prediction of the ability to predict outside of 50 m is relatively weak. In order to test the performance of the algorithmic models used in the current research, the performance of traditional machine learning and deep learning algorithms were compared with the models used in the research and obtained as shown in Table 3.

**Table 3:** Comparison of predictive performance of different algorithm models.

/	Research use model	Random forest	SVM	GBDT	XGBOOST	LSTM	GRU	Transformer
Accuracy (%)	96.2	92.1	91.3	88.4	90.3	94.3	90.4	89.7
Average percentage error	2.3	4.6	4.9	5.3	6	2.9	3.6	4.8
Root mean square error	3.6	4.6	5.1	6.5	6.2	5.4	3.9	4.6
Number of optimal solutions	20	10	13	9	16	17	12	9

From Table 3, it can be seen that the algorithmic model used in the study has the best prediction accuracy for metro data with the highest accuracy of 96.2%, which is 7.8% higher compared to the GBDT algorithmic model with the lowest accuracy. When comparing the change in prediction error, the algorithmic model used in the study has the lowest error value, with an average percentage error and root mean square error of 2.3 and 3.6, respectively, and the number of optimal solutions for the algorithmic model used in the study is higher in the dataset with 20 solutions compared to the other algorithmic models. This shows that the algorithmic model used in the study has better performance in predicting the vibration of underground.

## Discussion

With the acceleration of urbanization, many cities around the world are actively developing and expanding their metro systems to meet the growing demand for public transportation. As an efficient and environmentally friendly mode of urban transportation, metro plays an important role in alleviating urban traffic pressure and reducing carbon emissions. The vibration generated by subway operation will have an impact on the surrounding environment, such as affecting the structural safety of buildings and disturbing the lives of residents. With the advancement of technology, machine learning algorithms and database technologies are widely used in various fields. These technologies can process and analyze large amounts of data to discover complex relationships and patterns between data. In the prediction of vibration in subway environments, these techniques can help researchers predict vibration impacts more accurately and provide data support for the development of vibration mitigation measures and urban planning. Therefore this research addresses the metro transportation environment for research and analysis.

For this reason, when the actual measured and predicted values of track vibration are analyzed, the overall vibration value shows a gradient upward trend, and the changes of the predicted curve and the real curve basically coincide, but the simulated response acceleration at 0 m from the center distance is slightly lower than the actual response acceleration, which may be due to the interference caused by the operation and the operation of the vehicle in the actual measurement, and the error of the whole data measurement results. At the same time, some unevenness in the track can also cause measurement errors. In the measurement of different depths of the soil layer, the change of the measured value of different depths of the soil layer shows a tendency of rising and then falling, according to the curve change, it can be found that the peak value of acceleration gradually decreases after the depth of the ground increases, which indicates that the amplitude of the additive number shows a decreasing trend when the overall depth increases, which may be caused by the vibration propagation being affected. When comparing the loss function change of the algorithm, the trend of the algorithm loss function change increases with the increase of the iteration function, but the loss function value of the algorithm model tends to stabilize after reaching a certain value, which may be caused by the fact that the algorithm model begins to stabilize after the number of iterations reaches a certain value. In the data analysis of the actual measurement values of the algorithm model, it is found that the actual measurement results of the whole algorithm increase with the increase of the sample data values, and the change trend of the whole model becomes a gradient upward trend, but there is a decline in some data values, which may be due to the fact that the whole model will be affected by the measurement error when increasing the data measurement. When comparing the predicted value of the maximum vibration level of the ground surface with the real measured value, it is found that the difference between the actual measured value and the predicted value of the working condition is large, which may be due to the non-standardization of the operation during the measurement, and it may also be due to the bad effect of the data model used. When measuring and analyzing the horizontal distance, it is found that the actual measured values and the predicted values are in good agreement, which may be due to the fact that the horizontal distance is far away from the building when the horizontal distance is measured. To summarize, there are still some flaws in the current study, so the study will be followed by further research. When comparing the different conventional algorithmic models, the algorithmic model used in the study performs better in terms of performance and parameters, with the highest accuracy of 96.2 per cent, which is 7.8 per cent higher compared to the GBDT algorithmic model, which has the lowest accuracy. The mean percentage error and root mean square error were 2.3 and 3.6 respectively. This may be due to the fact that the algorithmic model used in the current study is a neural network model more suitable for the prediction of traffic vibration in a metro environment. The data model constructed in the study can better predict the vibration of the current complex underground traffic environment, and can achieve more and more effective underground environment analysis through more complex data response and regional prediction, which makes the construction and analysis of the current underground have a better guiding role. At the same time, the database technology used in the study can store more and more huge data, which also brings a better research direction for the lack of data of the current underground traffic environment.

## Conclusions

The experiment is the prediction of the subway traffic environment vibration model, through the introduction of the vibration prediction method, built a database model and the subway environment track prediction model, using machine learning algorithms combined with the database model of vibration data algorithmic analysis, to achieve the algorithmic model and the vibration model of the test, and finally through the experimental test of the real value and the predicted value of the situation. The experimental results show that changes in the depth of the surface will make the frequency range of the value follow the changes, while the prediction results of the vibration frequency range and the actual results compared to the error value is small basically match the actual vibration, the maximum acceleration value of the four different depths were 0.1, 0.04, 0.03, 0.02, the algorithm predicts the degree of coincidence of 92.57%, the whole prediction and the real value of an average difference of 1.4 dB, of which the maximum acceleration value was 0.1 dB. 1.4 dB, of which the maximum difference error value is 0.29%, the maximum error difference is 8.2%, and the approximate value is 6.2 dB, the four average values of the prediction in 40 m are relatively small 1.6 dB, and the average error value of the prediction ability between 40 and 100 m is 1.72 dB. It can be seen that the algorithm is able to achieve a high degree of coincidence in the prediction of the vibration model while the deviation value is being relatively small. Despite the good results obtained in this study, there are still many problems. Among them, the algorithm shows a weak ability in testing depths below 50 m, and the experimental data is not comprehensive enough is also a big problem. Therefore, the algorithm will be tested on more and more comprehensive data in the future, and the algorithm will be optimized for soil layers below 50 m depth.

## Data Availability

All data generated or analysed during this study are included in this published article.

## References

[1] Hong, T. K., Park, S. & Lee, J. Roles of subway speed and configuration on subway-induced seismic noises in an urban region. *J. Appl. Geophys.* **202**(32), 1232–1245 (2022).

[2] Zhou, X. Numerical analysis of influence of different track structures on vibration response of subway. *Therm. Sci.* **24**(1), 19–20 (2020).

[3] Huang, H., Zhao, M., Rong, Y., Sun, Y. & Xiao, X. Analysis of the vibration of the ground surface by using the layered soil: Viscoelastic euler beam model due to the moving load. *Math. Probl. Eng.* **21**(5), 652–659 (2021).

[4] Wang, S., Li, J., Luo, H. & Zhu, H. Damage identification in underground tunnel structures with wavelet based residual force vector. *Eng. Struct.* **178**(1), 506–520 (2019). 10.1016/j.engstruct.2018.10.021

[5] Ling, Y., Gu, J., Yang, T. Y., Liu, R. & Huang, Y. Serviceability assessment of subway induced vibration of a frame structure using FEM. *Struct. Eng. Mech.* **71**(2), 131–138 (2019).

[6] Dai, C. *et al.* Analysis of three-dimensional vibration characteristics of single-circle double-track subway tunnel under moving load. *Math. Probl. Eng.* **2021**(5), 2–14 (2021).

[7] Wang, L. *et al.* Time-frequency random approach for prediction of subway train-induced tunnel and ground vibrations. *Struct. Stab. Dyn.* **21**(7), 1–29 (2021).

[8] Liu, S. Measurement and analysis of vibration and noise in the ambient environment of metro. *Measurement* **163**(5), 10–24 (2020).

[9] Huang, H. Y., Zhao, M. J., Rong, Y., Sun, Y. & Xiao, X. Analysis of the vibration of the ground surface by using the layered soil: Viscoelastic euler beam model due to the moving load. *Math. Probl. Eng.* **4**, 2–15 (2021).

[10] Sheng, T. *et al.* Experimental study on the sandbag isolator of buildings for subway-induced vertical vibration and secondary air-borne noise. *Geotext. Geomembr.* **48**(4), 504–515 (2020). 10.1016/j.geotexmem.2020.02.008

[11] Wang, L., Han, Y., Zhu, Z. & Peng, H. Efficient time-frequency approach for prediction of subway train-induced tunnel and ground vibrations. *Proc. Inst. Mech. Eng. Part F J Rail Rapid Transit.* **236**(3), 288–301 (2022). 10.1177/09544097211020586

[12] Khalil, A. A., Metwally, K. G. & Ahmed, N. Z. Influence of rubber pads on vibration levels and structural behavior of subway tunnels. *J. Low Freq. Noise Vib. Active Control* **40**(3), 1493–1508 (2021). 10.1177/1461348420972831

[13] Yang, J. H., Lam, H. & An, Y. Development of a two-phase adaptive MCMC method for efficient Bayesian model updating of complex dynamic systems. *Eng. Struct.* **270**(11), 582–595 (2022).

[14] Santos, D. P. D., Gidro, G. D. M. S. & Carrazedo, R. A simplified numerical model for the assessment of vibration in subway lines with experimental validation. *Int. J. Struct. Stab. Dyn.* **21**(11), 10–43 (2021).

[15] Wang, L. *et al.* Time–frequency random approach for prediction of subway train-induced tunnel and ground vibrations. *Int. J. Struct. Stab. Dyn.* **21**(7), 10–39 (2021). 10.1142/S0219455421501017

[16] Huang, P., Ge, H. & Chen, Z. Rapid seismic damage evaluation of subway stations using machine learning techniques. *Comput. Methods* **20**(7), 134–139 (2022).

[17] Kushwaha, N. L. *et al.* Evaluation of data-driven hybrid machine learning algorithms for modelling daily reference evapotranspiration. *Atmosphere-Ocean* **60**(5), 5–26 (2022). 10.1080/07055900.2022.2087589

[18] Akar, O. & Gormus, E. T. Land use/land cover mapping from airborne hyperspectral images with machine learning algorithms and contextual information. *Geocarto Int.* **37**(16), 3963–3990 (2022). 10.1080/10106049.2021.1945149

[19] Musbah, H., Ali, G., Aly, H. H. & Little, T. A. Energy management using multi-criteria decision making and machine learning classification algorithms for intelligent system. *Electr. Power Syst. Res.* **203**(1), 1076–1085 (2022).

[20] Shim, J. G., Ryu, K. H., Lee, S. H. & Cho, E. Machine learning model for predicting the optimal depth of tracheal tube insertion in pediatric patients: A retrospective cohort study. *PLOS ONE* **16**(9), 541–562 (2021). 10.1371/journal.pone.0257069 PMC8412312 34473775

[21] Napoli, M. D., Martire, D. D., Bausilio, G. & Calcaterra, D. Rainfall-induced shallow landslide detachment, transit and runout susceptibility mapping by integrating machine learning techniques and gis-based approaches. *Water* **13**(4), 488–493 (2021). 10.3390/w13040488

[22] Tang, Z. *et al.* A data-informed analytical approach to human-scale greenway planning: Integrating multi-sourced urban data with machine learning algorithms. *Urban For. Urban Green.* **56**(14), 126–131 (2020).

[23] Rashid, M., Singh, H. & Goyal, V. The use of machine learning and deep learning algorithms in functional magnetic resonance imaging-A systematic review. *Expert Syst.* **37**(6), 352–384 (2020). 10.1111/exsy.12644

[24] Armstrong, D. J., Jevgenij, G. & Theodoros, D. Exoplanet validation with machine learning: 50 new validated kepler planets. *Mon. Not. R. Astron. Soc.* **504**(4), 5327–5344 (2020). 10.1093/mnras/staa2498

[25] Fang, Y. *et al.* ST-SIGMA: Spatio-temporal semantics and interaction graph aggregation for multi-agent perception and trajectory forecasting. *CAAI Trans. Intell. Technol.* **7**(4), 744–757 (2022). 10.1049/cit2.12145

[26] Guo, Y., Mustafaoglu, Z. & Koundal, D. Spam detection using bidirectional transformers and machine learning classifier algorithms. *J. Comput. Cogn. Eng.* **2**(1), 5–9 (2022).

